# An (Un)Expected Threat for a Regionally Near-Threatened Species: A Predation Case of a Persian Squirrel on an Insular Ecosystem

**DOI:** 10.3390/ani13010024

**Published:** 2022-12-21

**Authors:** Yiannis G. Zevgolis, Apostolos Christopoulos, Ioannis Ilias Kalargalis, Stylianos P. Zannetos, Iosif Botetzagias, Panayiotis G. Dimitrakopoulos

**Affiliations:** 1 Biodiversity Conservation Laboratory, Department of Environment, University of the Aegean, 81100 Mytilene, Greece; 2 Department of Zoology and Marine Biology, Faculty of Biology, National and Kapodistrian University of Athens, 15772 Athens, Greece; 3 Department of Cultural Technology and Communication, University of the Aegean, 81100 Mytilene, Greece; 4 Laboratory for Environmental Policy and Strategic Environment Management, Department of Environment, University of the Aegean, 81100 Mytilene, Greece; 5 Society for the Protection of Animals–KIVOTOS, 81100 Mytilene, Greece

**Keywords:** *Sciurus anomalus*, *Felis catus*, predation event, invasive species, islands, Lesvos

## Abstract

**Simple Summary:**

Among introduced alien species, one of the most widespread and successful predators on island ecosystems is the domestic cat. This opportunistic carnivore is considered responsible for numerous island species’ extinctions and species’ population declines, with small mammals having the higher predation risk among all species that are preyed upon. In this work, we present the first evidence, from the island of Lesvos, of a feral cat hunting, killing, and consuming a full-grown Persian squirrel. In the locality of the event, we further examined both squirrels’ and cats’ population densities based on these species’ home ranges. Results showed that the population of feral cats is almost fifteen times higher than that of squirrels, which raises concerns regarding the species’ conservation. Our observation reinforces the need for feral cat management to be prioritized for the conservation of this native and regionally near-threatened species.

**Abstract:**

One of the most successful predators on island ecosystems is the domestic cat, which is considered responsible for the decline of numerous species’ populations. This can be estimated by the analysis of cats’ dietary habits, yet prey identification is not always possible, and thus, in cases where precise prey identification is required, one of the most accurate methods derives from observing the hunting process. However, the cryptic nature of the feral cats and the constant vigilance of the species that are preyed upon make the observation process difficult, especially when the prey has a low population density. Here, we report for the first time such a case: a feral cat that has ambushed, killed, and consumed a regionally near-threatened species, the Persian squirrel. This incidental observation happened in the squirrel’s westernmost end of its distribution, the island of Lesvos, Greece. Due to the unexpectedness of the event, in the following days, we estimated both the squirrels’ and cats’ population density. Results showed that while the density of the squirrels is moderate, the population density of the feral cats is almost fifteen times higher. For this reason, management actions need to be taken in an effort to minimize the impacts of feral cats on the native species of the island.

## 1. Introduction

Island biodiversity is globally affected by a wide range of threats varying in both intensity and duration, most of which originate from or are facilitated by human presence [1], resulting in the largest proportion of historical and prehistorical species extinctions [2] appearing on islands. One of the main drivers of island species’ decline or even extinction, along with habitat degradation, damage, and destruction [3,4], is the introduction of alien predators [5]. Islands, along with the native species living there, are exceptionally vulnerable and lack adaptation to alien predators, as they generally have high endemism [6], low species richness and abundance [7], simplified antagonistic trophic interactions among species [8], few or no predator species [9], and frequently naive native species [10,11,12].

Among human-mediated introduced alien species, one of the most widespread and successful predators on island ecosystems is the domestic cat, *Felis catus* [13,14,15]. Since its domestication from the Near Eastern wildcat [16], *F. catus* has been introduced intentionally (to control rodent populations) or unintentionally (through transportation) to more than 179,000 small and medium-sized islands around the world [17]. On the Mediterranean islands, cats have been closely linked to the evolution of human society since 9500 years BP [18], while in the Aegean archipelago cats have been introduced during the Bronze Age [19]. Regardless of the species classification (pet, free-ranging, feral, or semi-feral), this opportunistic and generalist carnivore is considered responsible for numerous island species’ extinctions, at least 26% on a global scale [20] and population declines [17], including a variety of different taxa of reptiles and amphibians [12,21], especially in its feral form. This is the reason why the feral cat is considered a “superpredator” [22] in the trophic network of islands and is listed as one of the 100 worst invasive species in the world [23], and thus, their management is crucial for minimizing the effects on island wildlife [24]. 

The fauna’s risk of predation by *F. catus* can be estimated by studying the feeding ecology of this predator. Analyses of cats’ dietary habits showed that they mainly prey on small mammals, followed by birds, reptiles, amphibians, and insects [25,26]. However, the identification of preyed-upon species deriving from cats’ dietary studies is a demanding process which makes their successful completion quite difficult [27]. In terms of clear and precise prey identification, one of the most accurate methods derives from observing the hunting process directly. Such events are difficult to be observed, especially due to the cryptic nature of the feral cats, but also in cases where the prey is constantly vigilant, as a means to detect predators immediately.

One such species is a southwest Asian tree squirrel, the Persian squirrel (*Sciurus anomalus*). However, little is known about the threats and predators of Persian squirrels; there is only one study that reports the predation of this species by golden eagles or eagle owls [28]. In this study, we present the first photographic evidence, from the island of Lesvos, Greece, of a feral cat killing and partially consuming a full-grown Persian squirrel. Due to the unexpectedness of the event, we further proceeded in estimating the density of the Persian squirrels close to the area of the event and the cats’ population in the wider area. We also discuss the potential threats to the species arising from this observation, and we propose management measures regarding the feral cat population on the island.

## 2. Materials and Methods

### 2.1. Study Area and Species Information

**Figure 1:**
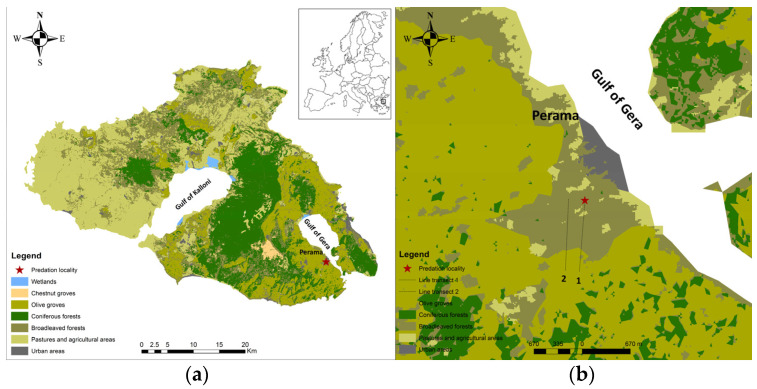
Distribution map of the main land cover types of the island of Lesvos along with (**a**) the locality of the incidental predation event and (**b**) the line transects.

Lesvos, the eighth-largest island of the Mediterranean basin and the third in Greece, with an area of 1632.8 km^2^, is located in the northeastern Aegean Sea. The island’s climate is typical Mediterranean, characterized by cool-moist winters and warm-dry summers [29]. Lesvos, a hilly island with a maximum altitude of 968 m, encompasses a variety of different habitat types, including extensive areas of Coniferous and Broadleaved forests mainly in the central part, traditional olive groves and grazing land in the eastern and southern part [30], while in its western and northern parts the island consists of shrubby vegetation and phrygana (Figure 1). Due to its vicinity to Asia Minor, the island’s diversity is influenced from the east, and thus, hosts a high number of biota, including the Persian squirrel.

The Persian squirrel (*Sciurus anomalus*) is the only known species of the family Sciuridae on the island of Lesvos as well as in the Eastern Mediterranean region. Its distribution extends North to Armenia, Georgia, and Azerbaijan, East to Iran and Iraq, South to Palestine, Jordan, Lebanon, and Syria, and West to Greece [31,32]. This species is widely distributed in Turkey, while Greece, particularly the island of Lesvos, is the westernmost end of its distribution. Despite population declines that have been observed, the last assessment of the species [32] has listed it as Least Concern. In Greece, the species is included in the National Red Data Book as Near Threatened [33], as it is threatened by habitat loss, expansion of the road network, and wildfires [34]. In other parts of its distribution range, threats include the deforestation of its habitat and poaching [32].

### 2.2. Estimation of Persian Squirrel Density

In the context of ongoing research since 2014 regarding the Persian squirrel’s presence on the island, we were on a field survey in the wider area of the village of Perama (39°2′35″ N, 26°30′16″ E) (Figure 1) in order to collect localities of the species.

In order to locate and record the squirrels’ density, after the predation incident, we used visual encounters in combination with the Distance Sampling Method [35]. Specifically, we created two line transects of 1 km each, covering as much woody vegetation in the area as possible; the total study area amounted to 10 ha. Transects did not overlap with each other and they had a vertical distance of 100 m between them. The first transect started from the point of the incident and had a southwest direction, while the second one was parallel to the first and had the same direction. The survey team consisted of one researcher at each transect, with observations made using binoculars, while transects had a width of 50 m: 25 m at each side. We chose these distances as approximations of the ‘home range’ of the species [36]. Observers’ distance from each individual was measured with a distance meter, and each transect was repeated two times, once every two days (27 October 2022 and 29 October 2022). Keeping in mind the ecological characteristics of the species, we conducted each survey during daylight hours from 09:00 to 11:00, and from 16:00 to 18:00, on sunny days with temperatures ranging from 18 to 25 °C. We estimated the Persian squirrels’ density by pooling the data from the line transects, using the equation D = N/(2 × W × L), where N is the number of individuals, W is the belt width to either side of the line, and L the length of the transect line.

We also obtained the habitat features of the area by using ArcGIS 10.2 software (ESRI Inc., Redlands, CA, USA). We extracted the total area of each land cover type and we calculated the mean tree cover density using data available from the COPERNICUS high-resolution layers [37,38,39].

### 2.3. Estimation of Cat Population

In order to obtain a relatively safe estimation of the cat population due to the fact that there are no data available for Lesvos Island, we conducted direct observations and interviewed local inhabitants based on a protocol created specifically for this purpose. Direct observations were carried out over the next few days (26, 28, and 30 October) by systematically searching both our study area (10 ha) and the wider region (approximately 200 ha) around the locality in which the incidence happened. The survey team consisted of two researchers, and observations were made using binoculars. Moreover, we interviewed people who occasionally had provided food to the cats, seeking information regarding the number of cats they had fed and the cats’ gender. In parallel, we reached out to the Lesvos-based animal welfare association, Kivotos Mytilene, to obtain primary data on the number of cats being neutered in the wider area.

## 3. Results

### 3.1. Detection of the Predation Incidence

**Figure 2:**
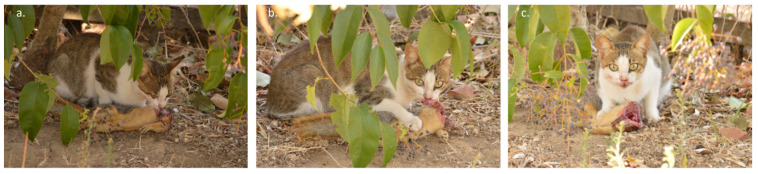
Photographic evidence, from the island of Lesvos, Greece, of a feral cat consuming a full-grown Persian squirrel: (**a**) the moment the cat came into our field of view, (**b**,**c**) the moment the cat realised our presence.

On 25 October 2022, at 16:09 h, during our field expedition, we detected a feral cat (39°2′14.17” N, 26°30′6.09” E) (Figure 1), approximately 20 m away from us, next to an olive tree trunk ready for an ambush (Figure 2). The day was sunny and the temperature was 22 °C. We stopped walking, we remained silent, and we observed the cat waiting to see what would happen next. Just a few seconds later an unexpected event happened: the cat managed to capture a full-grown Persian squirrel by the neck.

In an attempt to escape, the squirrel began to shake and made circular movements with his body to free himself, while at the same time trying to kick the cat with his hind legs. However, it was unable to escape, and the cat, holding the squirrel by the neck in his mouth, hid behind the trunk of the olive tree. Four minutes later, at 16:13 h, the cat came back into our field of view having decapitated the squirrel and began to consume it (Figure 2a). I.I.K. managed to take three photographs during the incident (Figure 2a–c). One minute later, and despite the fact that we remained silent throughout the incident and at a distance of more than 20 m, the cat realised our presence (Figure 2b,c), took the prey remains, and disappeared among the trees.

### 3.2. Persian Squirrel Density

The habitat of our 10 ha study area consists of a mosaic of olive groves (*Olea europaea*) with scattered broadleaf trees including pomegranate (*Punica granatum*), almond (*Prunus amygdalus*), walnut (*Juglans regia*), kermes oak (*Quercus coccifera*), and berries (*Rubus fruticosus*). There are also a few conifer trees (*Pinus brutia*) in the southeastern part of the study area. Specifically, the broadleaved trees had a density of 46.58%, covering most of our study area (77%), followed by the olive trees (16%) with a density of 52%, while the coniferous trees covered 6% of the area, having a density of 32%.

Persian squirrels’ population density was calculated to be 0.2 individuals per ha. In particular, in the first line transect we did not find any individual of the species during both days of our expedition, while in the second transect, on 29 October 2022, we found two individuals, on a walnut tree, at a distance of approximately 23 m, from our observation point.

### 3.3. Population of the Feral Cats

We identified, through direct observations and interviews, a total of 29 feral cats in our 10 ha study area. The feral cats directly observed were 12 females and 11 males, and for 6 cats it was not possible to determine their gender. Thus, a general estimation concerning the cats’ population density in the area, where the squirrels were observed, is 2.9 individuals per ha. Moreover, according to the animal welfare society of the island, in the wider region (200 ha) there is approximately a minimum absolute number of 32 cats of which 50% of the females were sterilized in 2022. However, their population is four to five times larger than the minimum number, i.e., approximately 120 to 150 animals (Kivotos Mytilene, personal communication, 2 November 2022).

## 4. Discussion

In this study, we report for the first time a feral cat killing and partially consuming a full-grown Persian squirrel. We consider that this incidental finding is particularly important, as it provides direct evidence that feral cats hunt, kill, and consume this species, constituting until now an unrecorded threat to it. The importance of this threat is amplified as the species is considered Near Threatened in Greece [33], while its population on the island of Lesvos is the only population of the species in the EU, and it is estimated from 500 to 3000 mature individuals [34]. 

The area in which the event was recorded is an ideal habitat for the species’ presence. Despite the fact that the Persian squirrel uses all habitats with trees, both cultivated and natural or semi-natural areas [36], tree and shrub species such as those found in the area are of great importance to the species not only as food sources but also because their trunk cavities provide suitable nest sites [40,41], pers. obs. 

On the one hand, this event was somewhat expected even though it was not recorded until now; the relatively moderate density of the species in this area, along with the feral cats’ population, which is almost fifteen times higher than the squirrels’, may in part, explain the observed killing event. After all, endemic and native island mammals are particularly vulnerable to cat predation because of their lack of anti-predatory behaviour [21]. Moreover, it is well known that the domestic cat is a mammalian predator of other squirrel species such as the Grey squirrel in America [42] but also to the Persian squirrel congeneric species, the Eurasian red squirrel [43]. In particular, predation incidents of the red squirrel, by feral cats, have been recorded by several authors [44,45,46] even though they did not exceed 10% of their studies’ sample size. However, it should be noted that in our case the density estimates of both squirrels and cats are a snapshot of their populations at a certain time and should not be taken as any form of precise population estimate.

On the other hand, the unexpectedness of this predation event derives from that in general, due to its morphometric, physiological, and behavioral traits, Persian squirrels are very difficult to be preyed upon; this is also true with other squirrel species that live in rural or natural areas [47,48]. Moreover, this species is diurnal and therefore, vigilance is one of its main characteristics to detect predators (pers. obs.). Another important element to reinforce the aforementioned fact is that cats were not introduced to Lesvos in recent years but during the Bronze Age [19]. This means that the Persian squirrel has learned to coexist with them, especially in the island’s rural areas.

While the reported observation demonstrates that feral cats can prey on Persian squirrels, a single observation does not provide any evidence as to the frequency, extent, or population impacts of such killings. Nevertheless, if a cat population persists in hunting within a small area the targeted animals could become highly stressed, and their population may collapse [49]. Moreover, considering the fact that cats are partially arboreal makes it difficult for squirrels to avoid being preyed upon by them. Another factor to be considered is that the cats’ home range probably overlaps with that of the Persian squirrel. Their home range can even reach 300 ha [50], with an average of 10 ha [49,51], while it is influenced mainly by the distribution of prey and less by the landscape characteristics; yet, it has also been related to gender, breeding season, sterilization, and age [52,53]. In parallel, unneutered cats have significantly larger home ranges than neutered cats [21] resulting in a negative effect on wildlife. In cases where these unneutered feral cats are in better states of physical fitness and/or leaner [54,55], it is likely that they will prey to a greater extent than a domestic cat. However, even if they are fed on a daily basis, their hunting instinct will not be suppressed [49]. They will continue to hunt, affecting not only the native species but also the native predators by out-competing them [56]. In our case, the exact population of unneutered cats in the focal area is relatively unknown, but we believe that it constitutes the largest proportion of their population. However, due to the fact that there are no scientific studies on the effect of sterilization on predation, we cannot speculate on this issue. 

Feral cats are known to have contributed significantly to island mammals’ population declines and extinctions [20]. Our incidental observation reinforces the need for feral cat management to be prioritized for the conservation of the Persian squirrel. An essential first step is to understand their impacts in order to acquire a complete view of this lurking threat for threatened and native species, such as the Persian squirrel. For this to be accomplished, cat dietary studies, as well as population studies regarding both species on the island, are considered crucial.

Feasible management measures for mitigating this issue, such as full-scale sterilization programs and community education [57,58], are considered crucial and should be adopted by the authorities in an effort to minimize the impacts of feral cats on the native species of the island.

## 5. Conclusions

In this study, we provided direct evidence of a feral cat ambushing, hunting, killing, and partially consuming a full-grown Persian squirrel, on the island of Lesvos, Greece. While the density of the squirrels in this area is moderate, the fact that the population of feral cats is almost fifteen times higher than that of squirrels is a cause of concern. This demonstrates the necessity of taking management actions for the conservation of this native species.

## Data Availability

The data that support the findings of this study are available from the corresponding author, (Y.G.Z.), upon reasonable request.
